# Stereotactic body radiation therapy for stage I medically operable non-small cell lung cancer

**DOI:** 10.1038/s41598-023-37643-7

**Published:** 2023-06-27

**Authors:** Esengul Kocak Uzel, Melisa Bagci Kilic, Hasan Morcali, Omer Uzel

**Affiliations:** 1 Department of Radiation Oncology, Health Sciences University Bakirkoy Dr. Sadi Konuk Training and Research Hospital, Zuhuratbaba District, Dr. Tevfik Saglam Street, 34147 Bakirkoy-Istanbul, Turkey; 2 Deparment of Radiation Oncology, Marmara University School of Medicine, 34899 Istanbul, Turkey; 3 Department of Radiation Oncology, Rumeli University Istanbul, Istanbul, Turkey; 4 Department of Radiation Oncology, Istanbul-Cerrahpasa University, Istanbul, Turkey

**Keywords:** Cancer, Diseases, Medical research, Oncology

## Abstract

Stereotactic ablative body radiation therapy (SBRT) has emerged as the standard treatment for inoperable patients with stage I non-small cell lung cancer (NSCLC). In the current study, we retrospectively analyzed a medically operable patient cohort with stage I NSCLC who refused surgery and subsequently underwent SBRT. Overall survival (OS) and progression-free survival (PFS) were calculated. Between April 2014 and July 2020, 55 patients were enrolled to the study. Forty (72.7%) patients were male, with a mean age of 69.85 ± 4.65 years (range 59–78 years). ECOG performance status were 0 and 1, except for one case. At the time of analysis, 8 deaths were observed. Of these, 25% (n = 2) died due to cardiac events, 12.5% (n = 1) due to pulmonary causes, 12.5% (n = 1) due to lung cancer-related causes, and the cause of death was unknown for 50% (n = 4). The pulmonary causes and cardiac events were not associated with radiation-induced toxicity. The median survival time was 34 months, with a range of 12 to 44 months. 2-year OS and PFS were 97% and 98%, 3-year OS and PFS were 82% and 77%, respectively. Treatment with SBRT was well tolerated and no grade 3 and 4 treatment-related adverse events were observed. SBRT seems to be a well- tolerated and effective alternative for patients with operable early-stage NSCLC.

## Introduction

Lung cancer is one of the most common cancer and deadliest thoracic malignancy for both men and women^1^. It is generally divided into non-small cell lung cancer (NSCLC) and small cell lung cancer histologically types. NSCLC is the most common type of lung cancer, accounting for approximately 80% to 85% of all lung cases^1^. Surgery remains the current recommendation for early-stage NSCLC; however, a significant proportion of the patients are considered inoperable due to various comorbidities^2^. Stereotactic Body Radiation Therapy (SBRT) uses small, highly focused, and accurate radiation beams to deliver potent doses and offers highly effective treatment that may be comparable to surgery in inoperable NSCLC patients, particularly with peripherally located tumors^4,4^. One of the main advantages of SBRT is the ability to deliver high doses to the target with better dose distributions while sparing normal structures with minimal radiation-induced toxicities. SBRT is well tolerated as an outpatient procedure, and has been reported to yield local control (LC) rates exceeding 90% in both medically operable and inoperable clinical stage I NSCLC^5^. Moreover, overall survival (OS) may be better after SBRT than after conventional radiation^6^.

In a combined analysis of randomized phase III STARS and ROSEL studies, which were terminated early due to slow recruitment, Chang et al. found higher OS with better tolerability with SBRT compared to surgery, suggesting that SBRT could be a treatment option in operable stage I NSCLC, while stimulating a debate around the subject^7^. In the current study, we analyzed overall survival (OS), progression-free survival (PFS), and treatment-related toxicity in stage I NSCLC patients who refused surgery and received SBRT.

## Materials and methods

### Patient selection

Routinely collected data of 55 previously untreated T1-2 NSCLC patients who were eligible for surgery, but refused surgery and therefore subsequently treated with SBRT between April 2014 and July 2020 were analyzed for the study. The clinical stage was determined according to the American Joint Committee on Cancer (AJCC) 8th Edition Lung Cancer Staging criteria, based on the review of computed tomography (CT) or positron emission tomography (PET/CT) with brain CT scan or magnetic resonance imaging (MRI). All patients underwent PET/CT imaging. When pathologic confirmation of cancer was not available, diagnosis was established by a multidisciplinary discussion using a combination of clinical and imaging findings. Empiric treatment criteria included tumor size > 0.5 cm, lesion with a solid component, lesion growth over time, and FDG avidity on PET/CT. All patients were treated by the same physician in various institutions. Adverse effects were reported according to the Common Terminology Criteria for Adverse Events, version 5 (CTCAEv5).

This study was approved by the institutional review board, the Ethical Committee of Istanbul Rumeli University (2020/11; 2.7.2020). The need to obtain informed patient consent was waived by the Ethical Committee of Istanbul Rumeli University due to its retrospective design. The study was performed in accordance to the relevant guidelines and regulations.

### Radiotherapy specifications

4D CT, breath hold CT and slow CT scans with 3 mm slice thickness were used in treatment planning. Internal target volume (ITV) was delineated based on these CTs. ITV plus 5–10 mm laterally and 5–20 mm craniocaudally based on planning CT was used for defining planning target volume (PTV). Treatment was given using volumetric modulated arc therapy (VMAT) on a Synergy® Linac (Elekta AB, Stockholm, Sweden), Truebeam Linac (Varian systems, USA) or TomoTherapy system (Accuray Inc., Sunnyvale, USA). In all patients, verification was done after patient positioning by daily cone beam CT or megavolt CT (MVCT) depending on the system available in each institution as described previously^8^.

**Figure 1:**
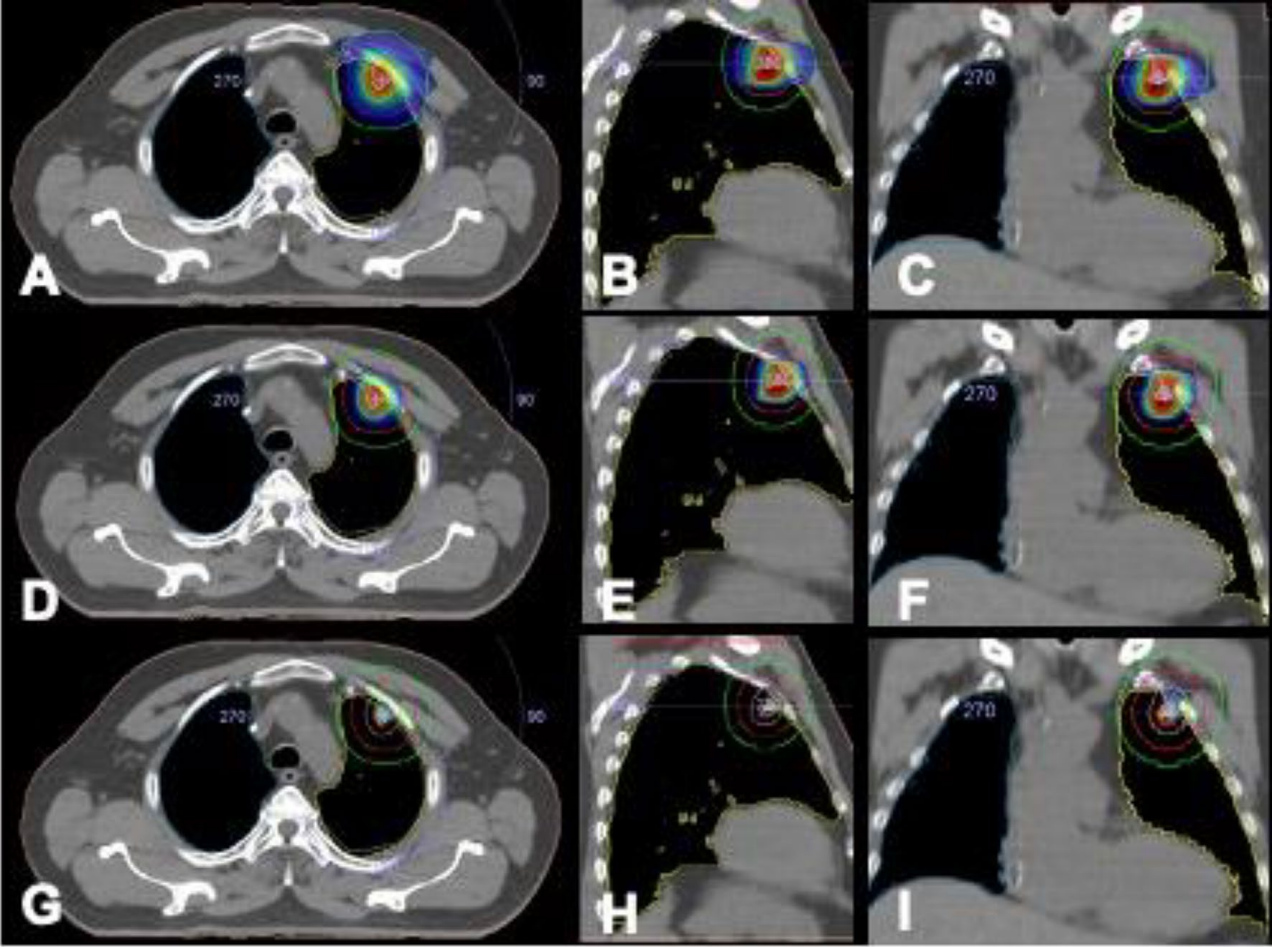
30% isodose line coverage (axial (**A**), sagital (**B**) and coronal (**C**) view), 50% isodose line coverage (axial (**D**), sagital (**E**) and coronal (**F**) view), 100% isodose line coverage (axial (**G**), sagital (**H**) and coronal (**I**) view).

**Figure 2:**
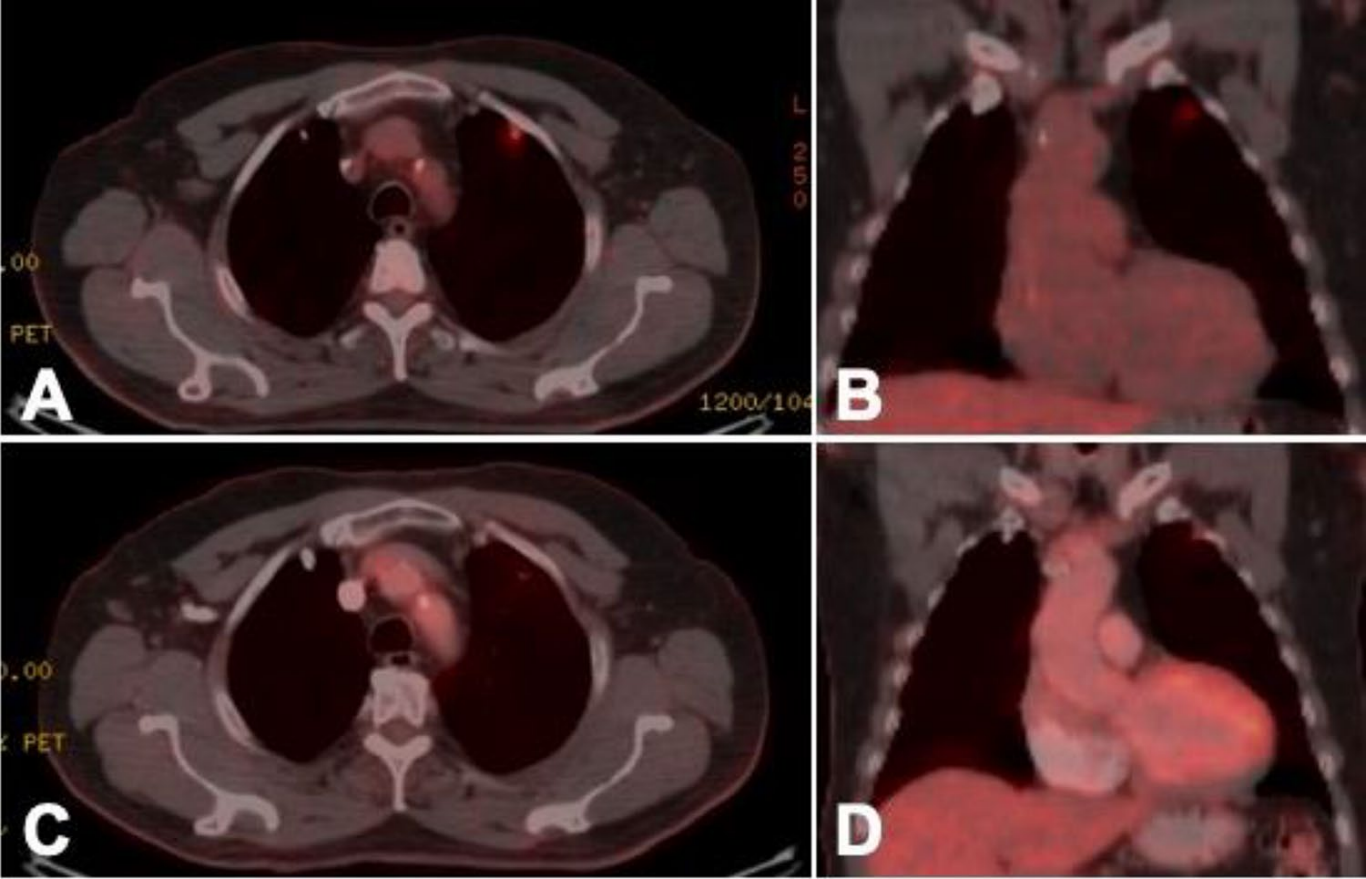
Pre-stereotactic body radiation therapy PET images. Axial (**A**) and coronal view (**B**) of the tumor center. Corresponding images in the 3-month follow up (**C,D**).

Fractionation schedules were at the discretion of the same physician according to the target site. Biological effectiveness dose (BED) > 100 Gy was administered in 3 or 5 fractions; and 8 fractions in patients with bigger tumors and central locations (within 2 cm of the proximal bronchial tree, heart, great vessels, trachea, or other mediastinal structures)^9^. BED values refer to the dose at the isocenter, with the 95% isodose encompassing the PTV. Each fractionation schedule had a minimum BED of 100 Gy (with an alpha/beta ratio of 10). An example of patient planning details and response PET/CT image was presented in Figs. 1 and 2.

### Statistical analysis

The Statistical Package for the Social Sciences (SPSS) program (version 16.0, SPSS Inc., Chicago, IL, USA) was used to analyse the data. Descriptive statistical methods (mean, standard deviation, median, frequency, percentage, minimum, maximum) were used while evaluating the study data. Normality of data distribution was evaluated with the Shapiro–Wilk test and graphical examinations. Mann–Whitney U test was used for comparisons between two groups of quantitative variables that did not show normal distribution. Fisher’s exact test and Fisher-Freeman-Halton exact test were used to compare qualitative data. Kaplan–Meier method was applied for survival analysis. Statistical significance was accepted as p < 0.05.

## Results

A total of 55 patients with a mean age of 69.8 ± 4.6 years were involved in the study. Forty patients were male (72.7%). The Eastern Cooperative Oncology Group (ECOG) performance status was assessed, revealing that among the participants, 30 (54.5%) had a score of 0, 24 (43.6%) had a score of 1, and only 1 patient (1.8%) had a score of 2.

Thirty-nine patients were smokers (70.9%). Baseline patient characteristics are presented in the Table 1.

**Table 1:** Patient and tumor characteristics.

		n (%)
Gender	Male	40 (72.7)
	Female	15 (27.3)
Age	Mean ± SD	69.85 ± 4.65
	Median (Min–Max)	70 (59–78)
Marital status	Married	20 (36.4)
	Not Married	35 (63.6)
Smoking status	No	16 (29.1)
	Yes	39 (70.9)
Pretreatment ECOG	0	30 (54.5)
	1	24 (43.6)
	2	1 (1.8)
Family lung cancer story	No	35 (63.6)
	Yes	20 (36.4)
COPD	No	49 (89.1)
	Yes	6 (10.9)
Biopsy	No	13 (23.6)
	Yes	42 (76.4)
Histology	Adenocarcinoma	24 (43.6)
	SCC	18 (32.7)
	No biopsy	13 (23.6)
Tumor location	Peripheral	35 (63.6)
	Central	20 (36.4)
Tumor location	RUL	15 (27.3)
	RML	3 (5.5)
	RLL	8 (14.5)
	LUL	22 (40.0)
	LLL	7 (12.7)
PTV (cm)	Mean ± SD	3.29 ± 1.41
	Median (Min–Max)	3.2 (1–5.3)
PTV (cc)	Mean ± SD	20.75 ± 21.69
	Median (Min–Max)	12.7 (1.4–86)
GTV (cc)	Mean ± SD	13.90 ± 15.38
	Median (Min–Max)	8.2 (0.5–65)
Technique	Breath hold	33 (60.0)
	Symmetry—4D CT	19 (34.5)
	Slow CT	3 (5.5)
Fraction	3	16 (29.1)
	5	31 (56.4)
	8	8 (14.5)

*ECOG* Eastern Cooperative Oncology Group Performance Status, *SCC* squamous cell carcinoma, *COPD* chronic obstructive pulmonary disease, *RUL* right upper lobe, *RML* right middle lobe, *RLL* right lower lobe, *LUL* left upper lobe, *LLL* left lower lobe, *PTV* planning target volume, *GTV* gross tumor volume, *CT* computed tomography.

Biopsy was performed in 76.4% (n = 42) of the cases, showing that 43.6% (n = 24) of the cases had adenocarcinoma, 32.7% (n = 18) had squamous cell carcinoma. Histological results were not available in 23.6% (n = 13) due to patient refusal. All patients had single lesions. Tumor location was peripheral in 63.6% (n = 35) and central in 36.4% (n = 20) of the cases. 40% (n = 22) of patients had left upper lobe, 12.7% (n = 7) left lower lobe, 27.3% (n = 15) right upper lobe, 5.5% (n = 3) right middle lobe, 14.5% (n = 8) had right lower lobe locations. Tumor characteristics are presented in Table 1.

The median PTV (in cm) was 3.2 cm (range 1 to 5.3 cm), the median the PTV (in cc) was 12.7 cc (range 1.4 to 86 cc), the GTV (in cc) was 8.2 cc (range 0.6 to 65 cc). The measurement represented by “cm” corresponds to the longest craniocaudal dimension. Total lung-PTV values of the cases ranged between 2806 and 7520, and the mean value was determined as 4302.56 ± 1225.83. Treatment planning was performed using breath hold technique in 60% (n = 33), 4DCT technique in 34.5% (n = 19), and slow CT technique in 5.5% (n = 3) (Table 1). Breath hold, Simetry-4DCT and slow CT techniques were similar in terms of efficacy and side effects profile.

**Figure 3:**
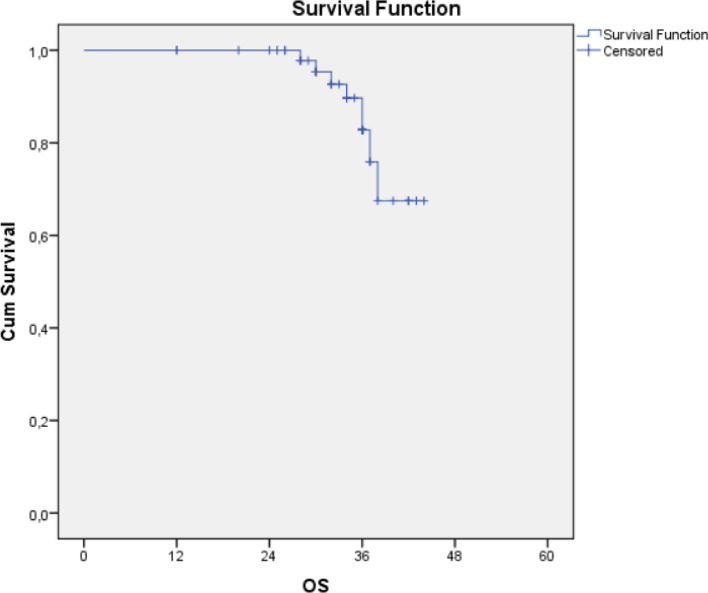
Overall survival analysis.

**Figure 4:**
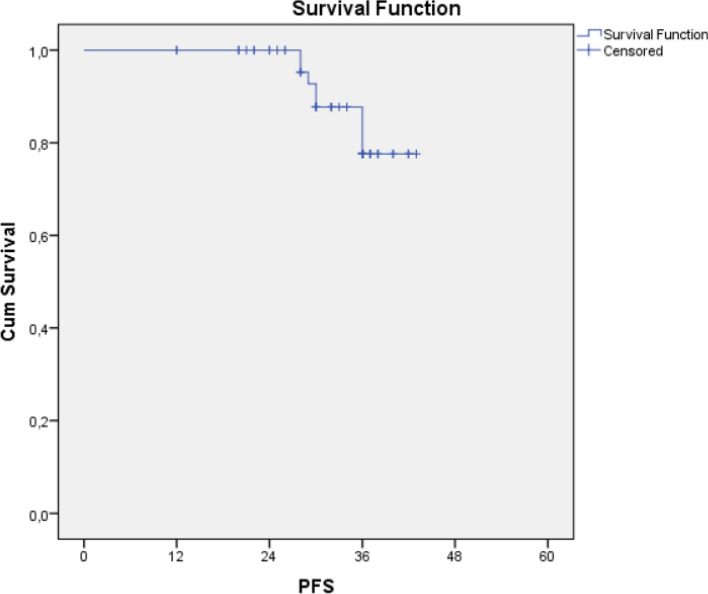
Progression free survival analysis.

The median survival time was 34 months, with a range from 12 to 44 months. Overall survival and PFS at 2 years were 97% and 98%, and 82% and 86% at 3 years, respectively (Figs. 3, 4). Until the time of the ultimate analysis, 8 deaths were observed, in 50% (n = 4) of which the cause was unknown, in 12.5% (n = 1) was lung cancer related, in 12.5% (n = 1) was due to pulmonary causes and in 25% (n = 2) was due to cardiac events. Two patients, aged 70 and 76, died from acute myocardial infarction, which is referred to as cardiac event. One patient died from shortness of breath resulting from COPD (Chronic Obstructive Pulmonary Disease), which is referred to as pulmonary cause, none of these three cases were not associated with radiation-induced toxicity. The results of the univariate analysis showed that pneumonia (%50 isodose line), chronic obstructive lung disease, total lung—PTV, and method of patient diagnosis were significantly associated with the 3-year OS. Patients with a total lung-PTV measurement less than 4400 cc had a significantly higher 3-year OS compared to those with more than 4400 cc (p = 0.013). However, no statistically significant differences were observed between the OS of the cases and the initial evaluation for pneumonia, tumor location, PTV, GTV, antibiotic prophylaxis, coverage values, and side effects. Furthermore, no significant effects of gender, age, marital status, smoking, ECOG performance status, or family history of lung cancer on survival were observed (p > 0.05). The results also showed that there were no statistically significant differences between the treatment techniques (Table 2).

**Table 2:** Univariate analysis of patient and treatment characteristics.

		3y OS (%)	*p*
Pneumonia (%50 isodose line)	No	97	0.000^a^^,^**
	Yes	67	
COPD	No	90	0.000^a^^,^**
	Yes	25	
Total lung—PTV (cc)	< 4400	73	0.013^b^^,^*
	≥ 4400	92	
Technique	Breath hold	86	0.273^c^
	Symmetry-4DCT	70	
	Slow CT	100	
Diagnosis	Cardiac follow-up	87	0.003^c^^,^**
	Interstitial lung disease follow-up	67	
	Check up	100	

^a^Fisher’s Exact Test.

^b^Mann Whitney U Test.

^c^Fisher Freeman Halton Test.

**p < 0.01.

None of the patients in our cohort experienced any grade 3 or 4 treatment-related adverse events, according to the CTCAEv5. Sixty percent (n = 33) of the cases did not have any side effects. Other patients reported grade 1 fatigue (18.2%, n = 10), grade 1 esophagitis (14.5%, n = 8), grade 1 chest wall pain (3.6%, n = 2), grade 1 hemorrhage (1.8%, n = 1) and grade 2 tracheal necrosis (1.8%, n = 1). Patients with central tumors experienced more esophagitis, hemorrhage, tracheal necrosis, and fatigue, whereas chest pain was reported more frequently in peripheral tumors. The majority of patients, 85.5% (n = 47), did not develop radiation pneumonia; however, 14.5% (n = 8) had symptomatic and radiologically confirmed grade 2 radiation pneumonia (Table 3).

**Table 3:** Adverse events with stereotactic body radiation therapy.

No	33 (60.0)
Radiation pneumonia*	8 (14.5)
Esophagitis	8 (14.5)
Chest wall pain	2 (3.6)
Hemorrhage	1 (1.8)
Trachea necrosis	1 (1.8)
Fatigue	10 (18.2)

*Symptomatic, radiation pneumonia.

## Discussion

The results of the current study suggest that SBRT might be a safe and effective treatment modality for medically operable NSCLC patients, with high rates of long-term disease control and with low rates of treatment-related adverse events. Additionally, we found that PFS and OS did not differ significantly by tumor size and tumor location, treatment technique, fractionation regimen, and patient characteristics except for COPD.

Surgery is the current standard treatment in early-stage NSCLC; however, a significant portion of patients are considered ineligible for surgery due to various reasons including advanced age, comorbidities and frailty. SBRT emerged as an alternative to surgery in these patients and its efficacy has been explored in many previous studies, showing 2- to 3-year LC rates of around 90% and 2- to 3-year overall survival rates ranging from 43 to 60%^3^. The convenience and non-invasiveness of SBRT together with the encouraging results from the inoperable patients naturally resulted in an interest to expand this treatment to all patients, despite the concerns that the good control rates seen in the inoperable patient population may not be applicable to medically operable patients who have a longer life expectancy^5^. Multicenter Japanese JCOG 0403 study included 164 patients (100 inoperable, 64 operable) and reported a 3-year OS of 59.9% (95% confidence interval 49.6–68.8%) and 76.5% (95% confidence interval 64.0–85.1%) in inoperable and operable groups, respectively with good tolerability (JCOG 0403)^10^. Single-arm phase 2 NRG Oncology Radiation Therapy Oncology Group 0618 study demonstrated that estimated 4-year primary tumor control and LC rate were both 96% (95% CI 83–100%) in operable early stage lung cancer patients^5^. An Italian retrospective trial also showed comparable OS in operable patients treated with surgery (lobar/sub-lobar resection) and with SBRT^8^. These findings including ours suggest that SBRT may be an effective alternative to surgery in operable patients.

Generally, candidate patients for SBRT are older with comorbidities compared to those undergoing surgery, this always favors surgery in survival outcomes. Therefore, comparisons from retrospective and population-based studies have significant limitations and are subject to biases. A decisive answer to whether outcomes from SBRT are comparable to surgery for stage I NSCLC would require large randomized studies. Nevertheless, randomized controlled ACOSOG Z4099^11^, STARS, ROSEL and SABRTooth^12^ trials all failed to achieve the predefined recruitment targets. Yet, among these studies, ROSEL and STARS had similar entry criteria, allowing a pooled analysis. In a such pooled analysis of 58 patients (31 SBRT and 27 surgery patients), Chang et al*.* reported that pooled estimated OS was 100% (95% CI 100–100) and 95% (95% CI 85–100) in the SBRT group, and 88% (95% CI 77–100) and 79% (95% CI 64–97) in the surgical group at 1 year and 3 years, respectively. In the SBRT group, recurrence-free survival at 3 years was 86% (95% CI 74–100), whereas 80% (65–97) in the surgery group (HR 0·69 [95% CI 0.21–2.29], log-rank p = 0.54)^7^. Recently, long-term results of the revised STARS trial, in which the SBRT group was re-accrued with a larger sample size of 80 patients, and compared to a protocol-specified propensity-matched cohort of patients who underwent video-assisted thoracoscopic surgery was published. Investigators demonstrated that OS was 91% (95% CI 85–98) at 3 years and 87% (79–95) at 5 years with SBRT, whereas 91% (95% CI 85–98) at 3 years and 84% (76–93) at 5 years with surgery, showing non-inferior long-term survival with SBRT compared to surgery^13^. Three phase III trials from North America are ongoing and are expected to provide valuable contribution on the SBRT vs surgery debate. Multicenter, randomized phase III the VALOR trial^14^ clinical trial will involve 670 patients comparing SBRT and surgery (lobectomy/segmentectomy) in stage I peripheral NSCLC. Another currently recruiting study, Stable-Mates trial^15^ has a planned sample size of 272 patients, and will compare sublobar resection and SBRT in high-risk patients. Thirdly, the Canadian radiotherapy LUSTRE trial is the only randomized phase III trial comparing SBRT with conventionally hypofractionated RT for the treatment of medically inoperable stage I NSCLC population^16^.

Surgical treatment allows ruling out occult lymph node involvement and performing pathological evaluation of disease at the same time, whereas SBRT relies on PET-CT assessment of nodal involvement and pre-treatment biopsy. On the other hand, previous reports demonstrated a low incidence of nodal relapse after treatment and no impact of histology on primary clinical outcomes^8^. Moreover, studies showed that SBRT have little effect on QoL and it is cost- effective, although cost-effectiveness results are controversial^17–19^. With the current data demonstrating the non-inferiority of SBRT in early stage NSCLC^13^, the number of patients whom SBRT could be offered would rise significantly^20,21^.

Regarding the relationship between BED and local control, for all treatment methods and schedules, the LC and survival rates were better with a BED of 100 Gy or more compared with less than 100 Gy^20^. Many studies showed that BED of less than 180 Gy was safe for stage I NSCLC, and the LC and OS rates in 5 years with a BED of 100 Gy or more were higher than the reported results for conventional radiotherapy. A previous studies showed that BED_10_ > 100 is related to favorable 3-year LC^22^, which might further increase with dose escalation protocols^23^. In another retrospective study, OS and LC were significantly greater in the SBRT group (48–52 Gy in 4–5 fractions, BED_10_ > 130 Gy) compared to matched patients treated with accelerated radiotherapy (48–60 Gy in 12–15 fractions, BED10ca. 80–90 Gy)^24–26^.

However, PFS and OS were similar regardless of the fractionation schedules in our study.

One of the main advantages of SBRT is the ability to deliver high doses to the target, while sparing normal tissues with minimal radiation-induced toxicities^25^. In this study, treatment was well-tolerated, without any grade 3 and 4 treatment-related AEs, in parallel to the previously reported very low rates of toxicity with SBRT. In our study, majority of patients were treated to 60 Gy in 5 fractions, and developed grade < 2 pneumonitis (14%) and very low rates of chest wall pain syndrome (3.6%). These treatment-related adverse event rates seem to be more favorable than early reports in the literature. For instance, an early a report of patients treated from 2004 to 2006 in Japan demonstrated a 29% rate of pneumonitis. However, more recent studies have reported similar low toxicity rates, such as rib fracture rates of 6.9%, chest wall syndrome rate of 8.3%, pneumonitis rate of 10.9%^27,28^. In regards to scanning protocols, there was no difference between Breath hold, Simetry-4DCT and Slow CT techniques from efficacy and side effects profile in our study. Therefore, SBRT seems an effective treatment and shows low rates of toxicity, when the appropriate number of fractions and techniques tailored for the patient.

Many retrospective series showed that comorbidities like COPD found to be one of major factor affecting survival in patients who had surgery or SBRT. In keeping with previous literature, we also found mortality rate of those with COPD was found to be significantly higher than those without COPD. However, we could not identify any prognostic factors associated with OS except for lung pathologies.

There are several limitations of this study. First, in the absence of randomized data, the study is subject to limitations and potential bias of the observational data. Second, over the last years, there is increasing reliance on clinical imaging for the diagnosis, prognosis, and treatment evaluation in early stage NSCLC patients high risk for biopsy^29^. Approximately one quarter of patients in the current study were treated without a biopsy confirmation, possibly increasing the LC rates. Nevertheless, in accordance with recent ASTRO SBRT guidelines for treatment in patients without pathologic confirmation, before SBRT a multidisciplinary approach was performed in patient evaluation utilizing the combination of all available clinical and imaging data in our cohort^30^.

In conclusion, SBRT might be an effective alternative with low rates of toxicity when appropriately tailored for early-stage operable NSCLC patients who refuse surgical treatment.

### Ethics approval

The current study was approved by the Ethical Committee of Istanbul Rumeli University (2020/11; 2.7.2020).

## Data Availability

The datasets used and/or analysed during the current study available from the corresponding author on reasonable request.

## References

[1] *Lung Cancer-Non-small Cell|Cancer.Net*. https://www.cancer.net/cancer-types/lung-cancer-non-small-cell/statistics%20last%20login%2031.01.2022.

[2] Robinson CG, Bradley JD (2010). The treatment of early-stage disease. Semin. Radiat. Oncol..

[3] Tandberg DJ, Tong BC, Ackerson BG, Kelsey CR (2018). Surgery versus stereotactic body radiation therapy for stage I non-small cell lung cancer: A comprehensive review. Cancer.

[4] Timmerman RD, Kavanagh BD, Cho LC, Papiez L, Xing L (2007). Stereotactic body radiation therapy in multiple organ sites. J. Clin. Oncol. Off. J. Am. Soc. Clin. Oncol..

[5] Timmerman RD (2018). Stereotactic body radiation therapy for operable early-stage lung cancer: Findings from the NRG oncology RTOG 0618 trial. JAMA Oncol..

[6] Palma D (2010). Impact of introducing stereotactic lung radiotherapy for elderly patients with stage I non-small-cell lung cancer: A population-based time-trend analysis. J. Clin. Oncol. Off. J. Am. Soc. Clin. Oncol..

[7] Chang JY (2015). Stereotactic ablative radiotherapy versus lobectomy for operable stage I non-small-cell lung cancer: A pooled analysis of two randomised trials. Lancet Oncol..

[8] Scotti V (2019). Stereotactic ablative radiotherapy as an alternative to lobectomy in patients with medically operable stage I NSCLC: A retrospective, multicenter analysis. Clin. Lung Cancer.

[9] Park HS, Harder EM, Mancini BR, Decker RH (2015). Central versus peripheral tumor location: Influence on survival, local control, and toxicity following stereotactic body radiotherapy for primary non-small-cell lung cancer. J. Thorac. Oncol..

[10] Nagata Y (2015). Prospective trial of stereotactic body radiation therapy for both operable and inoperable T1N0M0 non-small cell lung cancer: Japan Clinical Oncology Group Study JCOG0403. Int. J. Radiat. Oncol. Biol. Phys..

[11] Fernando HC, Timmerman R (2012). American College of Surgeons Oncology Group Z4099/Radiation Therapy Oncology Group 1021: A randomized study of sublobar resection compared with stereotactic body radiotherapy for high-risk stage I non-small cell lung cancer. J. Thorac. Cardiovasc. Surg..

[12] Franks KN (2020). SABRTooth: A randomised controlled feasibility study of stereotactic ablative radiotherapy (SABR) with surgery in patients with peripheral stage I nonsmall cell lung cancer considered to be at higher risk of complications from surgical resection. Eur. Respir. J..

[13] Chang JY, Mehran RJ, Feng L, Verma V, Liao Z, Welsh JW, Lin SH, O'Reilly MS, Jeter MD, Balter PA, McRae SE (2021). Stereotactic ablative radiotherapy for operable stage I non-small-cell lung cancer (revised STARS): Long-term results of a single-arm, prospective trial with prespecified comparison to surgery. Lancet Oncol..

[14] VA Office of Research and Development. *CSP #2005—Veterans Affairs Lung Cancer Surgery or Stereotactic Radiotherapy Trial (VALOR)*. https://clinicaltrials.gov/ct2/show/NCT02984761 (2023).

[15] Timmerman, R. *JoLT-Ca A Randomized Phase III Study of Sublobar Resection (SR) Versus Stereotactic Ablative Radiotherapy (SAbR) in High Risk Patients with Stage I Non-small Cell Lung Cancer (NSCLC), the STABLE-MATES Trial*. https://clinicaltrials.gov/ct2/show/NCT02468024 (2023). 10.1245/s10434-022-11584-3 35524087

[16] *Canadian Phase III Randomized Trial of Stereotactic Body Radiotherapy Versus Conventionally Hypofractionated Radiotherapy for Stage I, Medically Inoperable Non-small-cell Lung Cancer—Rationale and Protocol Design for the Ontario Clinical Oncology Group (OCOG)-LUSTRE Trial|Cochrane Library*. 10.1002/central/CN-01337269. 10.1016/j.cllc.2016.08.002 27876603

[17] Mitera G (2014). Cost-effectiveness analysis comparing conventional versus stereotactic body radiotherapy for surgically ineligible stage I non-small-cell lung cancer. J. Oncol. Pract..

[18] Lievens Y, Obyn C, Mertens A-S, Halewyck DV, Hulstaert F (2015). Stereotactic body radiotherapy for lung cancer: How much does it really cost?. J. Thorac. Oncol..

[19] Leaman-Alcibar O, Cigarral C, Déniz C, Romero-Palomar I, Navarro-Martin A (2021). Quality of life after stereotactic body radiation therapy versus video-assisted thoracic surgery in early stage non-small cell lung cancer. Is there enough data to make a recommendation?. J. Clin. Transl. Res..

[20] Onishi H (2007). Hypofractionated stereotactic radiotherapy (HypoFXSRT) for stage I non-small cell lung cancer: Updated results of 257 patients in a Japanese multi-institutional study. J. Thorac. Oncol. Off. Publ. Int. Assoc. Study Lung Cancer.

[21] Senan S, Palma DA, Lagerwaard FJ (2011). Stereotactic ablative radiotherapy for stage I NSCLC: Recent advances and controversies. J. Thorac. Dis..

[22] *Stereotactic Hypofractionated High-Dose Irradiation for Stage I Nonsmall Cell Lung Carcinoma: Clinical Outcomes in 245 Subjects in a Japanese Multiinstitutional Study*. https://pubmed.ncbi.nlm.nih.gov/15378503/. 10.1002/cncr.20539 15378503

[23] Alite F, Mahadevan A (2020). Dose escalation in the era of ablative lung irradiation: Is more dose better when it comes to delivery of lung stereotactic body radiation therapy?. Ann. Transl. Med..

[24] Chiang A (2016). A comparison between accelerated hypofractionation and stereotactic ablative radiotherapy (SABR) for early-stage non-small cell lung cancer (NSCLC): Results of a propensity score-matched analysis. Radiother. Oncol. J. Eur. Soc. Ther. Radiol. Oncol..

[25] Schonewolf CA (2019). Five-year long-term outcomes of stereotactic body radiation therapy for operable versus medically inoperable stage I non-small-cell lung cancer: Analysis by operability, fractionation regimen, tumor size, and tumor location. Clin. Lung Cancer.

[26] Yamashita H (2007). Exceptionally high incidence of symptomatic grade 2–5 radiation pneumonitis after stereotactic radiation therapy for lung tumors. Radiat. Oncol. Lond. Engl..

[27] Coroller TP (2014). Low incidence of chest wall pain with a risk-adapted lung stereotactic body radiation therapy approach using three or five fractions based on chest wall dosimetry. PLoS ONE.

[28] Borst GR (2009). Radiation pneumonitis in patients treated for malignant pulmonary lesions with hypofractionated radiation therapy. Radiother. Oncol. J. Eur. Soc. Ther. Radiol. Oncol..

[29] Hasan S, Colonias A, Mickus T, VanDeusen M, Wegner RE (2018). Image-based management of empiric lung stereotactic body radiotherapy (SBRT) without biopsy: Predictors from a 10-year single institution experience. Thorac. Cancer.

[30] Videtic GMM (2017). Stereotactic body radiation therapy for early-stage non-small cell lung cancer: Executive summary of an ASTRO evidence-based guideline. Pract. Radiat. Oncol..

